# Die ambivalente Rolle der Spiritualität für die Erklärung von Verschwörungsglauben und Demonstrationsbereitschaft im Kontext der COVID-19-Pandemie

**DOI:** 10.1007/s41682-022-00134-z

**Published:** 2022-11-11

**Authors:** Rebecca Endtricht

**Affiliations:** Abteilung für Kriminologie, Universität Hamburg, Hamburg, Deutschland

**Keywords:** Spirituality, Esotericism, Conspiracy belief, COVID-19, Preventive measures, Protest, Spiritualität, Esoterik, Aberglaube, Verschwörungsglaube, COVID-19, Corona-Demonstrationen

## Abstract

Im Kontext der COVID-19-Pandemie gewinnen zunehmend Gruppen an Sichtbarkeit, die sich als Anker in der Problembewältigung präsentieren und vermeintliche Lösungsansätze zum Umgang mit der Pandemie zur Verfügung stellen. In Teilen solcher Strömungen zeigt sich ein Hang zum Verschwörungsglauben sowie zu abergläubischen und esoterischen Deutungsmustern, die in den Protesten gegen Maßnahmen zur Eindämmung der Pandemie vermehrt zum Ausdruck kommen. Die Vermengung dieser Phänomene in einer gemeinsamen Protestbewegung wirft die Fragen auf, wie diese zusammenhängen und welche Rolle dabei religiöse Zugehörigkeiten und spirituelle Weltanschauungen einnehmen. In der vorliegenden Studie werden spirituelle Welt- und Wertebilder differenziert und religionsunabhängig erfasst, um diesen Fragen nachzugehen. Es lassen sich empirisch zwei Formen der Spiritualität, die *aktive* und *passive Spiritualität* voneinander trennen, die sich in den Dimensionen Weltbild und Ethik, Wertekanon und Sinnempfinden teils diametral gegenüberstehen. Es zeigen sich unterschiedliche Effekte dieser beiden Spiritualitätsformen auf die Offenheit gegenüber alternativen und esoterischen Welterklärungen sowie auf die Ausprägung von Skepsis gegenüber der Wissenschaft. Die spirituellen Elemente tragen auch zur Klärung des Glaubens an Verschwörungserzählungen, der Unterstützung von Corona-Demonstrationen und einer diesbezüglichen Teilnahmebereitschaft bei. Dabei wirkt die *aktive Spiritualität* als Schutzfaktor und die *passive Spiritualität* demgegenüber als verstärkender (Risiko‑)Faktor für die Auftretenswahrscheinlichkeit von sowohl Verschwörungsglauben als auch Protestbereitschaft. Insgesamt zeigt sich, dass eine mehrdimensionale Erfassung von Spiritualität zur Erklärung von verschwörungstheoretischen und protestrelevanten Einstellungen beiträgt. Auf diese Weise können zudem bisherige ambivalente Befunde der Forschung zu den Effekten von Spiritualität als Resilienz- bzw. Risikofaktor für solche Einstellungen und Verhaltensbereitschaften ausdifferenziert werden.

## Abstract (en)

In the course of the COVID-19-pandemic, groups that present themselves as anchors in problem solving and provide supposed solutions for dealing with insecurities are becoming increasingly visible. There seems to be a tendency for conspiracy belief and esoteric attitudes, which are increasingly being expressed in the protests against measures to contain the pandemic. This raises the question of the role of religious affiliations and spiritual worldviews within such groups. In the present study, spirituality is differentiated and measured independently of religion in order to shed light on these questions. The results show two forms of spirituality that differ in the dimensions of worldviews and ethics, values and meaning of life. Both forms can be found in religious as well as non-religious groups. Analyses show different effects of these forms of spirituality on the openness towards alternative and esoteric explanations of the world as well as a skepticism towards science. Additionally, all spiritual elements also contribute to clarifying the belief in conspiracy stories, the support of COVID-related demonstrations and the willingness to participate in these demonstrations. *Active spirituality* acts as a protective factor and *passive spirituality* as a reinforcing (risk) factor for these phenomena. Overall, this multi-dimensional measurement of spiritual worldviews can explain attitudes relevant to conspiracy belief and protests, and in this way can break down the previous ambivalent effects of spirituality as a resilience vs. risk factor for such attitudes.

## Einleitung

In den im letzten Jahr entstandenen Protesten gegen die Maßnahmen zur Eindämmung der COVID-19-Pandemie waren innerhalb der protestierenden Gruppen immer wieder Mischungen aus Verschwörungsnarrativen, abergläubischen und esoterischen Deutungsmustern, bis hin zu antidemokratischen Haltungen zu finden. In Teilen dieser ideologischen und quasi-religiösen Strömungen scheint ein Hang zu Verschwörungsmythen und rechtem Gedankengut zu bestehen (vgl. van Prooijen und Douglas 2017; Wiedemann 2016), der im Kontext der Corona-Pandemie öffentlich an Sichtbarkeit gewonnen hat. Die Vermengung solcher Phänomene in einer gemeinsamen Protestbewegung wirft die Frage auf, inwieweit diese zusammenhängen und welche gemeinsamen Anknüpfungspunkte sie untereinander haben. In der sozialwissenschaftlichen Forschung zu diesem Thema herrscht Einigkeit darüber, dass die Pandemie als Krisensituation zu einer vermehrten Unsicherheit und zu einem Gefühl des Kontrollverlusts innerhalb der Bevölkerung führt, in dessen Folge es zu einem vermehrten Bedürfnis nach Sinn und Kontingenzbewältigung seitens der Individuen kommt (vgl. Friedrichs 2021; Endrass et al. 2021). Bei der Suche nach Erklärungen im Rahmen der Bewältigung solcher negativer Gefühle kommen unter anderem Verschwörungsnarrative zum Tragen, die den Bedürfnissen nach leicht verständlichen, klaren Informationen, Aufklärung über Hintergründe und nicht zuletzt auch nach einer Verortung von Schuldigen entgegen kommen.

In diesem Kontext wird vermehrt der Zusammenhang des Glaubens an Verschwörungsnarrative mit Religion und religiösen Überzeugungen diskutiert, die allerdings eine ambivalente Rolle einzunehmen scheinen. Einerseits bieten Religionen einen Ansatzpunkt zur Kontingenzbewältigung, vor allem im Rückgriff auf gefestigte, klare Weltanschauungen und die Verfügbarkeit einer religiösen Gemeinschaft. Andererseits wird je nach religiöser Haltung und Einbettung aber auch von Zusammenhängen mit konservativen, regressiven und dichotomen („gut“ gegen „böse“) Einstellungen berichtet, die mit einem höheren Risiko für den Glauben an Verschwörungsnarrative einhergehen. Fraglich ist jedoch, inwieweit spezifische (religiöse) Einstellungen insbesondere in der pandemischen Situation zu einem erhöhten Verschwörungsglauben führen und welche Auswirkungen diese in Bezug auf die Einschätzung konkreter Maßnahmen zur Handhabung der Pandemie oder auch hinsichtlich der Bereitschaft zum Protest gegen solche Maßnahmen (im Rahmen sog. „Corona-Demonstrationen“) haben.

Die Rolle von Glaube und Religiosität in der Erklärung solcher Einstellungen wurde in der Forschung bislang primär im Hinblick auf Weltreligionen wie dem Christentum oder dem Islam betrachtet. Dabei bleibt allerdings unklar, welche Aspekte von Religion, neben der faktischen Religionszugehörigkeit, hier tatsächlich einen Einfluss haben. Nichtreligiöse Glaubensformen, d. h. alternative Weltansichten und Spiritualität, finden in diesem Forschungsfeld bislang zudem kaum Beachtung. Angesichts des hohen Anteils konfessionsloser, nichtreligiöser Menschen in Deutschland erscheint dies erstaunlich, insbesondere vor dem Hintergrund, dass auch diese sich nicht in einem ideologiefreien Raum bewegen, sondern sich anhand ihrer Welt- und Wertebilder mitunter durchaus an glaubensbezogene Haltungen koppeln (vgl. Pickel und Jaeckel 2015).

In der vorliegenden Studie werden Glaubensformen, Weltanschauungen und Spiritualität religionsübergreifend erfasst und analysiert. Ziel ist die Untersuchung der Frage, inwiefern konkrete spirituelle bzw. religiöse Haltungen mit einem als problematisch eingeschätzten Glauben an bestimmte übernatürliche bzw. parapsychologische Phänomene einhergehen – unabhängig von der Zugehörigkeit zu einer bestimmten Religion. Auf dieser Basis wird sodann ein Zusammenhang zu der pandemischen gesellschaftlichen Situation hergestellt. Es werden Einflüsse von Formen der Spiritualität, von Esoterik und Wissenschaftsskepsis auf COVID-bezogene Verschwörungstheorien einerseits sowie die Einstellung zu Corona-Demonstrationen andererseits analysiert. Die Untersuchungen werden auf Basis der Daten einer Bevölkerungsbefragung aus dem Sommer 2021 durchgeführt, in der spirituelle Weltanschauungen und Glaubensformen sowie die Haltung zur Pandemie, u. a. im Rahmen ihrer wahrgenommenen Gründe und Folgen, umfassend erhoben wurden.

## (Nicht‑)Religiosität und Spiritualität

### Definitionen und Konzepte

Der Begriff der Spiritualität wird häufig im engen Zusammenhang mit der Definition religiöser Überzeugungen genannt, wonach Spiritualität Bestandteil aller Religionen sei (vgl. Demmrich und Huber 2019). Dabei wird Religion als eine übergeordnete Entität betrachtet, die sich dadurch auszeichnet, dass sie formalisiert und in institutionalisierten Gemeinschaften organisiert ist. Allerdings ist die „Frage nach der Definition ihres Gegenstandes in der Religionssoziologie von Anfang an mit einer auffälligen Unsicherheit verbunden“ (Pollack 2017, S. 8). Dieser Umstand führt auch dazu, dass Religion und Spiritualität selten hinreichend klar empirisch konzeptualisiert werden.

Im Rahmen der Definition bzw. Eingrenzung dessen, was Religion und Religiosität sind, kann zunächst der Begriff „*religio *im Sinne von Gottesverehrung“ (Seewald 2020, S. 18, Hervorhebung im Original) herangezogen werden. Auf dieser Basis beschreiben entsprechende Definitionsansätze Religionen anhand ihrer zentralen Merkmale, die sich um einen Gottesglauben drehen und Rituale, Traditionen und Gemeinschaften beinhalten, die sich auf einen gemeinsamen Gott beziehen. Nach den Ansätzen der Intentionsforschung werden darüber hinaus aber auch individuelle, von einer wie auch immer gearteten höheren Instanz losgelöste, Erfahrungen mit dem Religionsbegriff umfasst. Demnach „handelt es sich überall dort um Religion, wo Intentionen über den Horizont des Alltagslebens hinausführen“ und sich auf etwas richten, das mit weltlichen Erklärungen nicht ausreichend beschrieben werden kann (Pollack 2017, S. 28). Auf diese Weise können auch individuelle Erfahrungen, „die an und für sich gar nicht einer ausgeprägten und gelebten ‚Religion‘ oder religiösen Tradition zugehören“ (Waardenburg 1984, zit. nach ebenda, S. 28), in theoretische Konzepte einbezogen werden. Schon in dieser ersten Zusammenfassung zentraler Definitionsbestandteile wird die dort implizierte enge Verknüpfung von transzendentalen und immanenten Bestandteilen von Glauben deutlich: während sich transzendentale Inhalte des Glaubens außerhalb der Lebensrealität von Menschen bewegen, macht „die gleichzeitige Bezugnahme auf immanente Vorstellungsgehalte und Praktiken das Transzendente präsent und damit erfahrbar und kommunikabel“ (ebenda, S. 27). So umfassten beispielsweise schon die fünf religiösen Dimensionen von Glock (1962) – Erfahrungen, Rituale, Ideologie/Glaubenssätze, Wissensdimensionen, Konsequenzen – solche Komponenten, die sich auf individuelle Erfahrungen und Interpretationen des Erlebten im Kontext des subjektiven Weltbildes einer Person beziehen. Transzendentale Elemente sind somit inhärenter Bestandteil von Religionen, gehen aber gleichzeitig über anhand ihrer organisierten Form vorgenommenen Definitionen und Eingrenzungen hinaus (vgl. Seewald 2020).

Ein solches Hinausgehen über organisierte Religionen führt zur Betrachtung von Spiritualität als Ausprägung des subjektiven Weltbildes einer Person. Grundlegend wird Spiritualität, u. a. auch von der WHO, als „Reflexion der Erfahrungen verstanden, die im Umgang mit existenziellen Fragen gemacht werden“ (Utsch 2018a, S. 31f.). Dabei werden Verbindungen zur Innen- und Außenwelt eines Individuums hergestellt, die je nach (wissenschaftlicher) Betrachtungsweise unterschiedliche Komponenten beinhalten. Laut Beneschs (1990) *spirituellen Weltanschauungen* sind dies die fünf Dimensionen Weltbild, Menschenbild, Sinnorientierung, Wertekanon sowie Moral/Ethik, anhand derer sich der spirituelle Glaube konkreter fassen lässt. Während sich die Dimensionen Menschenbild, Wertekanon und Moral/Ethik maßgeblich auf das Zusammenleben von Menschen beziehen, beinhalten die Dimensionen Weltbild und Sinnorientierung auch Vorstellungen, die über die physisch erlebte Welt hinaus gehen können, beispielsweise anhand von Vorstellungen über höhere Mächte oder zu übernatürlichen Zusammenhängen im Universum. Alle fünf Dimensionen gemeinsam bilden somit multiple Facetten persönlicher und sozialer Haltungen ab. Eine derartige Verknüpfung solch breit gefasster Spiritualitätsbestandteile findet sich unter anderem auch im dreidimensionalen Modell der Spiritualität von Bucher (2014). Dieses beinhaltet transzendentale Verbindungen zu einem spirituellen Wesen, immanente Verbindungen (z. B. zum Universum, der Natur oder der sozialen Umwelt) sowie die Verbindung zum Selbst. Personale und soziale, transzendentale und immanente Komponenten müssen in der empirischen Erfassung spiritueller Formen demnach gemeinsam gedacht werden. Die beiden beschriebenen Grundmodelle sollen daher in den folgenden Untersuchungen als konzeptionelle Basis für die Messung spiritueller Weltanschauungen dienen. Da für die Konzeption einer solchen Spiritualitätsskala hier ein explorativer Ansatz gewählt wird, wird dabei zunächst versucht, dasjenige Modell mit der breiteren Auffächerung, also das fünfdimensionale Modell spiritueller Weltanschauungen von Bucher, empirisch nachzustellen.

### Zur empirischen Messung von Spiritualität

Zur Problematik der validen Erfassung der Spiritualität merken Streib und Klein (2013) an, dass Spiritualität im allgemeinen Verständnis oft gleichgesetzt wird mit Erfahrungen, die sich purer Rationalität entziehen und dabei in ihrer empirischen Bedeutung ungenau bleiben (vgl. auch Pickel und Jaeckel 2015). Die Erfassung von Religiosität und Spiritualität geht in quantitativen Erhebungen zunächst oft miteinander einher, indem schlicht summarisch nach der Selbsteinschätzung der Religiosität bzw. Spiritualität gefragt wird („Als wie religiös/spirituell würden Sie sich selbst bezeichnen?“). Problematisch ist hier, dass aus Sicht der Forschenden unklar bleibt, was Befragte mit diesen Begriffen verbinden und warum sie sich auf einer solchen Basis als (nicht) religiös bzw. spirituell einschätzen (vgl. Cragun et al. 2016; Friedrichs 2021). Alternativ wird daher in vielen religionsbezogenen Untersuchungen die religiöse Praxis erfragt, z. B. anhand der Häufigkeit des Betens, des Besuchs eines Gotteshauses oder auch anhand der Ausprägung des Glaubens an religiöse Schriften oder Regeln (z. B. Siegers und Jedinger 2020), woraus auf eine stärker objektivierbare Ausprägung der Religiosität geschlossen werden soll. Ein zentrales Problem solcher Messungen liegt im impliziten Schluss von Form, Ausprägung und Intensität religiöser Komponenten auf die religiöse und spirituelle Weltanschauung (Pollack 2017, S‑17–18).

Darüber hinaus werden Haltungen von Personen, die sich keiner Religion zugehörig fühlen, oft gar nicht oder nur unzureichend erfasst (z. B. aufgrund von Filterführungen im Messinstrument). „Konfessionslosigkeit ist nun aber nicht zwangsläufig mit Atheismus gleichzusetzen“ (Pickel und Jaeckel 2015, S. 4). Einstellungsbefragungen, die sich mit dieser Personengruppe näher befassen, zeigen, dass sich ein großer Teil der Menschen, die sich keiner institutionalisierten Religion zuordnen, bei genauerer Nachfrage in anderer Form als gläubig, spirituell oder offen für alternative Welterklärungen zeigt (vgl. z. B. Krafft 2019). Daher kann aus einer Konfessionslosigkeit nicht automatisch darauf geschlossen werden, dass Glaubenskonstrukte per se abgelehnt werden. So zeigen jüngere Studien, dass Religionen in ihrer institutionalisierten Form für eine wachsende Anzahl von Menschen zunehmend uninteressant werden, deren Sinnsuche sich stattdessen auf den privaten Bereich verlagert und somit in anderer Form ausprägt und manifestiert (vgl. Endler 2019; Pickel und Jaeckel 2015). Da in Deutschland zu dieser Personengruppe zwischenzeitlich knapp 30 % der Bevölkerung zählen (vgl. Pickel 2020), ist dies ein nicht zu vernachlässigender Faktor in der Frage nach der Verbreitung spiritueller Weltanschauungen in der Bevölkerung.

Seit einigen Jahren finden sich vermehrt Ansätze, die religiöse und spirituelle Einstellungen konkreter erfassen wollen. Die Skala zur Zentralität der Religiosität (Huber 2003; Huber und Huber 2012) geht in diesem Sinne über die oben kritisierten Messungen hinaus. Sie zieht fünf Indikatoren zur Messung heran, darunter ideologische und intellektuelle Komponenten sowie Erfahrungen mit Gott bzw. etwas Göttlichem. Auch wenn hier erneut die Spiritualität als gesondertes Konstrukt nicht explizit benannt wird, stellen die Inhalte doch einen deutlichen Bezug zur individuellen Spiritualität einer Person her. Darauf aufbauend diskutieren Demmrich und Huber (2019, S. 3), ob eine ethische Dimension als sechste Dimension der Zentralitätsskala hinzugefügt werden müsste, um zu erfassen, ob auch „non-religious or atheist individuals report an orientation toward values in their everyday lives that can be explicitly reported or ethically defined as spiritual“. Auch Cragun und Kolleg*innen (2016) operationalisieren mit ihrer *Nonreligious-nonspiritual scale *spirituelle Erfahrungen detaillierter. Ihr Erhebungsinstrument umfasst Meinungen und Aussagen bezüglich des Lebens nach dem Tod, einer höheren Macht sowie zu übernatürlichen Erfahrungen, die sich vom weltlichen, körperlichen Erleben einer Person abheben. In Bezug auf die eingangs genannten fünf zentralen Dimensionen der Weltanschauung von Benesch bleiben hier allerdings die Dimensionen Sinnorientierung sowie Werte, Moral und Ethik weiterhin außen vor.

King und Hicks (2021) befassen sich diesbezüglich ausführlicher mit dem Sinn und den Zielen (*meaning* und *purpose)* des Lebens, die wiederum eng mit den Werten und dem Weltbild einer Person zusammenhängen: „Worldviews are overarching belief structures that provide a sense of how the world works. Worldviews help people answer the fundamental questions of life“ (ebenda, S. 571–72). Weiter helfen die moralischen und ethischen Ansichten und individuellen Werte dabei, aus Erlebnissen und Erfahrungen ein subjektives Sinnempfinden abzuleiten, das je nach Ausprägung und Interpretation in das breitere spirituelle Weltbild einer Person eingebettet ist.

Derartige Operationalisierungen und bisherige Ansätze für Messinstrumente sollen hier aufgegriffen, ausgebaut und auf ihre praktische Anwendbarkeit überprüft werden. Dabei sollen unterschiedliche Aspekte spiritueller Weltanschauungen erhoben werden, um mögliche Muster bzw. Formen der Spiritualität zu identifizieren, die sich anhand theoretisch postulierter Dimensionen definieren, vor allem aber auch differenzieren lassen. Ziel ist die Untersuchung der Fragen, welche Dimensionen der Spiritualität und welche zwischen diesen Dimensionen bestehende Bezüge tatsächlich empirisch nachweisbar sind, wie sie sich aufeinander beziehen und nicht zuletzt, wie sie in Relation zur Religion stehen. Daraus abgeleitet lautet die erste Forschungsfrage:

1. Welche Formen der Spiritualität gibt es?
  - Wie lassen sie sich anhand der Dimensionen Weltbild, Menschenbild, Sinnorientierung, Wertekanon sowie Moral und Ethik definieren und interpretieren?
  - Gibt es Unterschiede im Auftreten der Spiritualitätsformen zwischen Personen mit und ohne Religionszugehörigkeit?

## Die ambivalente Rolle der Spiritualität: „Alternative Spiritualität“ und Verschwörungsglaube

In der Religionsforschung herrscht zunächst Einigkeit in der Annahme, dass Religiosität und Spiritualität überwiegend dazu beitragen, Halt zu geben und Unsicherheiten zu verringern. Diese Annahme der Kontingenzbewältigung wurde bereits in vielen Studien bestätigt, bezieht sich aber – wie bereits ausgeführt – in der Regel auf den Aspekt der Religiosität. Allerdings zeigen beispielsweise Analysen aus dem Hoffnungsbarometer (Krafft 2019) durchgehend positive Zusammenhänge zwischen Gefühlen wie Hoffnung und Mitgefühl einerseits sowie Religiosität und Spiritualität andererseits. Spirituelle Komponenten können demnach dabei helfen, Sinn in einer säkularisierten Welt zu finden und so z. B. anhand der Harmoniesuche in Gemeinschaften oder des Aushaltens von Ungerechtigkeiten sichtbar werden. Spiritualität wird insofern als Coping-Ressource verstanden, die sich positiv auf die Gesundheit und Lebenszufriedenheit auswirken kann. Sie erweist sich „[…] als Moderator, der in Stresssituationen zur Belastungsbewältigung aktiviert werden kann und zu gezielter Problemlösung, kognitiver Umstrukturierung, Relativierung unerreichbarer Ziele oder zur emotionalen Unterstützung und Selbstaufwertung beiträgt“ (Utsch 2016, S. 1156). In diesem Sinne konnte in einer Meta-Analyse gezeigt werden, „dass Menschen mit religiösen und spirituellen Bewältigungsmechanismen ein höheres körperliches und emotionales Wohlbefinden sowie eine höhere Lebenszufriedenheit zeigen, weniger zu Alkohol greifen, weniger interpersonelle Probleme aufweisen, mehr soziale Unterstützung erhalten und allgemein besser mit schwierigen Umständen umgehen können“ (Pfeifer 2018, S. 188; vgl. Ano und Vasconcelles 2005). Als Mechanismen gelten u. a. positive Selbstwahrnehmungen, Möglichkeiten zur Emotionsregulation, gesteigerte Kontrollgefühle sowie ein daraus folgendes geringeres Stressempfinden (vgl. Updegraff et al. 2008; King und Hicks 2021).

Wenn aber (religiöse) Spiritualität einen solch positiven Einfluss aufweist, warum und unter welchen Bedingungen kann sie negative Effekte entfalten, wie sie sich u. a. aktuell im Kontext der Corona-Pandemie in bestimmten Gesellschaftsbereichen vermehrt zeigen? Es gibt hierzu einige theoretische und konzeptionelle Überlegungen, die mögliche Erklärungswege aufzeigen. So geht Baierl (2020) davon aus, dass Spiritualität prinzipiell sowohl Resilienz- als auch Risikofaktor für das persönliche Wohlbefinden von Jugendlichen sein kann. Den Unterschied machen dabei Rituale, die zentral zur Strukturierung des eigenen Glaubens, der Weltanschauung und letztendlich auch der Selbstwirksamkeit sind. Diese können je nach Rigidität der zugrunde liegenden Regeln auch vermehrt Druck und Unsicherheiten auslösen und den Umgang mit belastenden Situationen zusätzlich erschweren. Extreme spirituelle und religiöse Einstellungen können außerdem wirken, wenn sie die „Sehnsucht nach Gewissheit“ (Utsch 2018b, S. 4; vgl. Pargament 2002; von Gontard 2013) und Klarheit angesichts unübersichtlicher Alternativen stillen. Das Bedürfnis nach Angstbewältigung und Kontrolle resultiert dann in alternativen Sinn- und Weltdeutungen, die eine Handlungsalternative zur Bewältigung solcher Situationen liefern können – „Je ausgelieferter sich jemand fühlt, desto eher ist er/sie bereit, im Chaos eine Struktur zu erkennen“ (Endrass et al. 2021, S. 115, vgl. Roose 2020). Ein solches Erkennen von Struktur schlägt sich sodann mitunter in konkretisierten Ausdrucksformen spiritueller Überzeugungen nieder, die zur Bewältigung bestimmter (Krisen‑)Situationen beitragen können. Der Glaube an eine kosmische Ordnung hilft auf diese Weise dabei, Krisen sinnhaft umzudeuten und ein Kontrollgefühl wiederzuerlangen (Steppacher 2021; vgl. Douglas et al. 2019). Problematisch wird dies, wenn sich Personen auf Basis ihrer „transzendenten (Wahrheits‑)Ansprüche“ (Hidalgo 2020, S. 90) nicht mit der Realität zufriedengeben und Haltungen oder gar Handlungsalternativen in Betracht ziehen, die sich gegen gesellschaftliche bzw. politische Zustände oder Akteure richten (vgl. van Prooijen und Douglas 2017).

Derartige Handlungsalternativen werden von den Begriffen *Aberglaube* und *Esoterik* umfasst. Diese Formen der „alternativen Spiritualität“ (Endler 2019) drücken sich durch Praktiken und Rituale wie Wahrsagen, Tarot oder Pendeln aus, die auf dem prinzipiellen Glauben an Übernatürliches basieren und auf diese Weise ihren Ausdruck in konkreten, spirituell konnotierten Handlungen finden. Sie beruhen auf vermeintlich sicherem alternativen Wissen und sollen dabei helfen, eine übernatürliche Erkenntnis oder Erlösung zu erlangen (vgl. ebenda; Schäfer und Frei 2021). „Westliche Esoterik“ beinhaltet insofern „besonderes Wissen, das (…) durch Selbsterfahrung vermittelt werden kann und mithilfe dessen eine übernatürliche Erkenntnis oder Erlösung aus der menschlich-materiellen Welt angestrebt wird.“ (von Stuckrad 2004, S. 88). Aberglaube hingegen meint „Rituale und Sinnfindungen, die nicht direkt erfahren, sondern durch eine weitere Person vermittelt“ werden (Schließler et al. 2020, S. 286) – etwa durch die Praxis der Wahrsagerei. Durch solche Praktiken, die in ähnlicher Form bisweilen auch in Religionen zu finden sind, „nimmt der Mensch Kontakt zu diesen [übermenschlichen] Mächten auf oder gewinnt Zugang zu ihnen“ (Pollack 2017, S. 15). So können Komponenten der alternativen Spiritualität als empirisches Verbindungsglied zwischen spirituellen Überzeugungen und konkreten, „weltlichen“ Handlungen dienen und mutmaßlich die in der bisherigen Forschung häufig beobachtete Ambivalenz der Folgen verschiedener spiritueller Glaubensaspekte inhaltlich aufschlüsseln und erklärbar machen.

Als dritte Ausprägung alternativer Spiritualität wird neben Aberglauben und Esoterik vermehrt eine ablehnende Haltung gegenüber etablierten Wissenschaften, die sog. Wissenschaftsskepsis, in entsprechende Konzepte und Analysen einbezogen. Rizeq und Kolleg*innen (2021) gehen davon aus, dass Verschwörungsglaube, der Glaube an Übernatürliches und Wissenschaftsskepsis drei Komponenten einer zugrundeliegenden „contaminated mindware“ sind. Diese Faktoren führen gemeinsam zu einer Fehlwahrnehmung und -interpretation von Fakten und einer Überschätzung der Relevanz eigener, intuitiver Erlebnisse. Da auch die Wissenschaftsskepsis, wie Aberglaube und Esoterik, spirituelle Annahmen mit der Wahrnehmung weltlicher Anknüpfungspunkte verbindet (z. B. im Rahmen der Ablehnung bestimmter medizinischer Behandlungen), wird diese Komponente hier ebenfalls als Indikator für alternative Spiritualität aufgefasst. In einer entsprechenden Analyse vor dem Hintergrund der Pandemie zeigt sich, dass Aberglaube nur einen indirekten Effekt auf die Akzeptanz der Maßnahmen zur Eindämmung der Pandemie hat, der über den Glauben an COVID-Verschwörungstheorien mediiert wird. Wissenschaftsskepsis hingegen wirkt unabhängig davon direkt auf die Ablehnung der Corona-Maßnahmen (Hartmann und Müller 2022). In diesem Zusammenhang hat sich auch der Begriff „Conspirituality“ (Ward und Voas 2011) entwickelt, der auf Erkenntnissen beruht, wonach bei Personen mit esoterischen Haltungen vermehrt eine Verschwörungsmentalität festzustellen ist (Schäfer und Frei 2021, S. 398; Schließler et al. 2020, S. 285).

Weitere Studien, die sich mit der pandemischen Situation befassen, zeigen zunächst, dass 10 bis 30 % der Menschen in Deutschland eine Affinität bzw. eine Zustimmung zu COVID-Verschwörungsnarrativen ausdrücken (vgl. z. B. IfD Allensbach 2022; Wetzels und Brettfeld 2022; Roose 2020). Anhängern von Verschwörungstheorien und abergläubischen bzw. esoterischen Personen ist in diesem Kontext gemein, dass sie eher eine „querulatorische Persönlichkeit“ sowie ein ausgeprägtes Schwarz-Weiß-Denken aufweisen und eher zu Misstrauen neigen (Endrass et al. 2021, S. 113). Dies geht einher mit dem Denken in Vorurteilen und dem Bedürfnis nach einfachen Erklärungen. Hier spielen auch generalisierte Kontrollüberzeugungen eine wesentliche Rolle. Gehen Personen in hohem Maße davon aus, für ihr Leben und ihr Schicksal selbst verantwortlich zu sein (internale Kontrollüberzeugung), scheint dies mit einer erhöhten Akzeptanz von Maßnahmen zur Eindämmung der Pandemie einherzugehen, während Personen mit einer externalen Kontrollüberzeugung, die glauben, ihr Leben sei vor allem durch Schicksal oder höhere Mächte beeinflusst, sich kritischer gegenüber den Maßnahmen zeigen (Hartmann und Müller 2022). Diese Ergebnisse zeigten eine enge Verbindung zwischen Verschwörungs- und Aberglauben und deren Funktion auf. Beide dienen in der Regel dazu, Struktur und Sinn im Leben zu erkennen und etwas zu konstruieren, an dem man sich bei Unsicherheit festhalten kann: „Was immer dieser sein mag, immerhin gibt es einen Sinn. Aus dieser Sicht erscheint das Verschwörungsdenken weniger als Kritik denn als Rechtfertigungsmuster“ (Schäfer und Frei 2021, S. 400–401).

Auch in Bezug auf das gesellschaftliche und politische System als Ganzes zeigt sich, dass esoterische, abergläubische und wissenschaftsskeptische Elemente mit einer Ablehnung gesellschaftlicher Institutionen, der Ablehnung politischer Akteure und Parteien, vermehrter Intoleranz gegenüber Minderheiten sowie insbesondere auch der Ablehnung der geltenden Corona-Maßnahmen und einem erhöhten Protestpotenzial zusammenhängen (vgl. van Prooijen et al. 2022; Schließler et al. 2020; Grande et al. 2021; Wetzels und Brettfeld 2022; Nachtwey et al. 2020).

Es kann zusammengefasst werden, dass die soziologisch wie psychologisch relevante Trennlinie mit Bezug auf Spiritualität und Religiosität keineswegs zwischen religiösen einerseits und konfessionslosen Personen andererseits zu verlaufen scheint, sondern vielmehr zwischen Menschen, die „Kontingenzbewusstsein und Offenheit in ihr (…) Selbst- und Weltverhältnis integriert haben“ und solchen, die auf Basis ihrer spirituellen Überzeugungen soziale, gesellschaftliche und politische Mechanismen in Frage stellen – „gleichgültig, ob sie nun gläubig sind oder nicht“ (Straub 2016, S. 112). Die ambivalente Rolle spiritueller Weltanschauungen ergibt sich in diesem Sinne aus einer konstruktiven vs. destruktiven bzw. funktionalen vs. dysfunktionalen Ausprägung solcher Sinndeutungen, die entsprechend unterschiedliche Handlungsalternativen für das Individuum verfügbar und erstrebenswert machen (vgl. Friedrichs 2021; Pickel und Yendell 2022). Im Rahmen dieser Ambivalenz ist bisher allerdings noch unklar, wie spirituelle Weltanschauungen mit den hier genannten Komponenten der alternativen Spiritualität zusammenhängen, welche Handlungsalternativen also je nach Ausprägung des spirituellen Glaubens für das Individuum verfügbar erscheinen. Mit Blick auf die gesellschaftlichen Herausforderungen der Corona-Pandemie folgt daraus auch die Frage, inwiefern sie ein Risiko- oder Schutzpotenzial für problematische gesellschaftsrelevante Haltungen und Handlungen darstellen. So könnten beispielsweise Anknüpfungen an Sinnfindungspotenziale eines Aberglaubens Personen davor bewahren, Sinn in Verschwörungsnarrativen zu suchen (vgl. Endrass et al. 2021). Da aber „Verschwörungserzählungen als Hilfskonstruktion für esoterische Überzeugungen dienen“ (Schließler et al. 2020, S. 296) können, ist auch ein umgekehrter, also verstärkender Effekt zwischen einem bestimmten Sinnempfinden über Aberglauben einerseits und Verschwörungsglauben andererseits gleichfalls vorstellbar. Bei allen hier betrachteten Dimensionen und Faktoren scheint somit entscheidend zu sein, mit welchem Fokus der spirituelle Glaube von Personen tatsächlich umgesetzt wird. Daraus ergeben sich die Forschungsfragen 2 und 3:

2. In welchem Bezug stehen identifizierbare unterschiedliche Spiritualitätsformen zu Aberglauben, Esoterik und Wissenschaftsskepsis? Zeigen sich insoweit unterschiedliche Effekte von Formen der Spiritualität als Resilienz- vs. Risikofaktor?
3. Wie stellen sich die Einstellung von Personen mit verschiedenen spirituellen Weltanschauungen zu Corona-Verschwörungsnarrativen und Corona-Demonstrationen dar?
  - Wie stehen Personen je nach spiritueller Ausprägung zu Corona-Verschwörungserzählungen und zu Corona-Demonstrationen?
  - Welche Rolle spielen dabei die Komponenten der alternativen Spiritualität?

## Methodik und Operationalisierung

Die Daten für die vorliegenden Untersuchungen wurden im Rahmen eines Forschungsprojekts erhoben, das sich, eingebettet im Forschungsverbund MOTRA („Monitoringsystem und Transferplattform Radikalisierung“), mit der Untersuchung politischer Einstellungen und Formen von Extremismen befasst (Brettfeld et al. 2021). Im Kontext dieses Vorhabens spielen auch Formen möglicher religiös konnotierter politischer Extremismen eine besondere Rolle. Teil dieses Vorhabens ist auch die vorliegende standardisierte Befragung über ein Online Access Panel des Marktforschungsunternehmens respondi. Die Datenerhebung fand vom 26.07. bis 03.08.2021 per Online-Fragebogen statt, der etwa 25 min Bearbeitungszeit umfasste. N = 1216 Personen ab 16 Jahren nahmen an der Umfrage teil, wobei eine Quotenstichprobe zur gleichmäßigen Erfassung aller Altersgruppen und Geschlechter zur Anwendung kam. Von diesem Sample geht ein Subsample von *n* = 633 Personen, bei denen die hier relevanten Variablen erhoben wurden, in die im Folgenden durchgeführten Analysen ein. In dieser Analysestichprobe sind mit einem Anteil von 56 % etwas mehr Männer als Frauen vertreten. Zudem sind jüngere Personen in der Altersgruppe zwischen 16 und 39 Jahren mit 45 % im Vergleich zur Gesamtbevölkerung Deutschlands überrepräsentiert. Auch ein Bildungsbias ist festzustellen. 63 % der Befragten geben an, das Abitur oder einen Hochschulabschluss zu haben.

**Figure 1:**
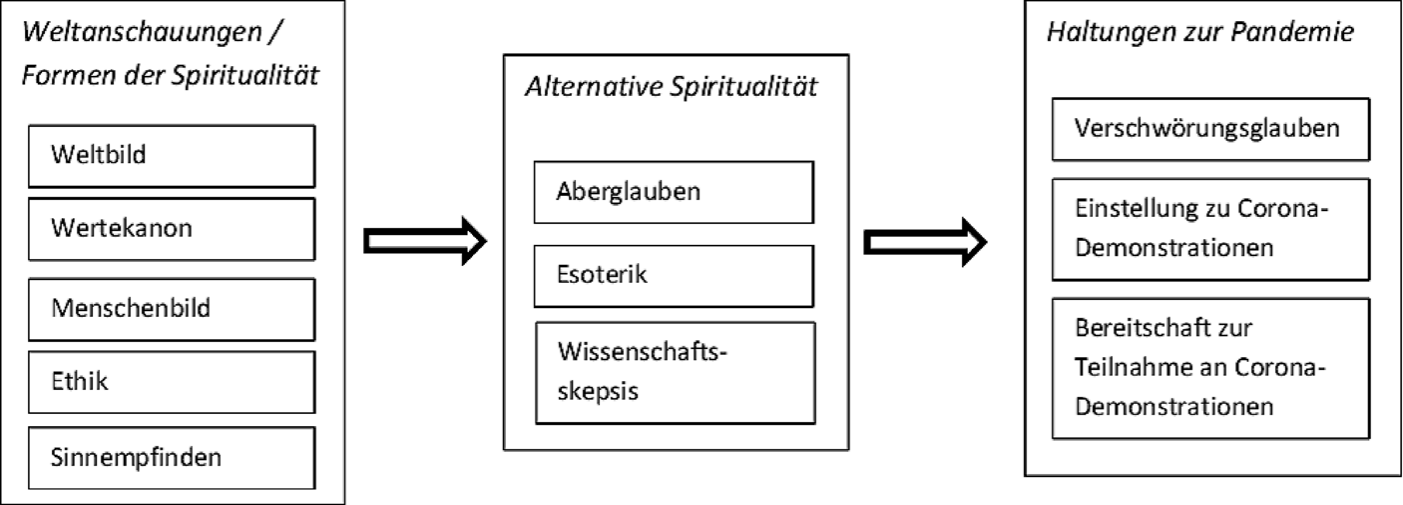

Zentrale Variablen sind zunächst die Weltanschauungen, auch als Formen der Spiritualität bezeichnet, sowie die Komponenten der alternativen Spiritualität. Hier werden Zusammenhänge zwischen diesen Aspekten von Spiritualität geprüft. Daran anknüpfend wird weiter untersucht, wie diese Variablen mit dem Glauben an Corona-bezogene Verschwörungsnarrative sowie mit Einstellungen zu den Corona-Demonstrationen zusammenhängen. Die Begrifflichkeiten und die Bestandteile der Konstrukte sind in Abb. 1 als Übersicht dargestellt.

Um verschiedene *Formen der Spiritualität *zu identifizieren, wurde nach Sichtung des einschlägigen Forschungsstandes, wie oben beschrieben, aus einem Item-Pool von über 100 Items eine Auswahl von 42 Items getroffen, die die theoretisch relevanten fünf Dimensionen der Spiritualität abbilden sollten. Der Item-Pool stellte sich zusammen aus Operationalisierungen der Spiritualität, die teilweise in bisherigen Fragebögen verwendet wurden (u. a. ALLBUS (Diekmann et al. 2013); WHO Quality of Life Questionnaire (Angermeyer et al. 2002); SBI-15R (Holland et al. 1998)). Zudem wurden für darüber hinaus relevant erscheinende Inhalte, die in qualitativen Studien herausgestellt wurden (insb. Demmrich und Huber 2019; Steensland et al. 2018), auch neue Items konstruiert. Alle verwendeten Items wurden auf einer 4‑stufigen Likert-Skala abgefragt.

Die *alternative Spiritualität* wurde über Inhalte aus den Bereichen Aberglaube, Esoterik und Wissenschaftsskepsis erhoben. Bezüglich des *Aberglaubens* kam ein Instrument zur Erfassung übernatürlicher Erfahrungen zur Anwendung. Die Befragten wurden gebeten anzugeben, ob sie sechs dargestellte Ereignisse selbst erlebt oder miterlebt haben. Hierbei handelte es sich um Ereignisse wie einen Kontakt zu Geistern, Kommunikation mit Engeln oder das Erleben einer Wunderheilung (operationalisiert auf Basis einer qualitativen Studie von Steppacher 2021). Als Antwortkategorien standen „ja“, „nein“ und „bin mir nicht sicher“ zur Verfügung. Inhaltlich kann davon ausgegangen werden, dass Personen, die die letztgenannte Kategorie wählten, zumindest eine Offenheit für derartige Erlebnisse zeigen. Sie werden deshalb für die weitere Skalenkonstruktion als Zustimmung recodiert.^1^ Die Skala wurde als Summenskala mit einem Wertebereich von 0 (kein Erlebnis) bis 6 (alle Ereignisse wurden erlebt) konstruiert (M = 1,19, SD = 1,66; Cronbach’s Alpha = 0,80). Es zeigt sich, wie erwartet, eine schiefe Verteilung: 51 % der Befragten geben an, keines dieser Ereignisse erlebt zu haben.

*Esoterikglaube* wurde anhand von fünf Items erfasst, die den Glauben an die Wirksamkeit verschiedener Handlungen oder Dinge, z. B. Horoskope, Wahrsagen oder Pendeln, abfragte (angelehnt an die Erfassung aus dem ALLBUS; Diekmann et al. 2013). Die Befragten wurden gebeten, jeweils auf einer Skala von 1 „glaube überhaupt nicht daran“ bis 4 „glaube voll und ganz daran“ diese esoterischen Werkzeuge in Bezug auf ihre eingeschätzte Wirksamkeit zu beurteilen. Die auf dieser Grundlage gebildete Mittelwertskala weist einen Mittelwert von 1,74 auf (SD = 0,76; Cronbach’s Alpha = 0,89).

*Wissenschaftsskepsis *als drittes Element alternativer Spiritualität wurde über drei Items gemessen, die sich auf wissenschaftliche Erkenntnisse bzw. Grundlagen und deren Bewertung beziehen und die in vergleichbarer Form in weiteren aktuellen Studien verwendet werden (vgl. Schließler et al. 2020; Grande et al. 2021; Nachtwey et al. 2020). Hierzu zählt z. B. das Item „Wir sollten uns bei der Behandlung von Krankheiten nicht auf die Schulmedizin verlassen“. Auch hier konnte die Zustimmung bzw. Ablehnung auf einer Skala von 1 („stimme gar nicht zu“) bis 4 („stimme völlig zu“) angegeben werden. Die drei Items weisen mit Cronbach’s Alpha = 0,66 eine akzeptable interne Konsistenz für eine Skalenbildung auf (M = 2,27, SD = 0,73).

Als abhängige Variablen werden eine Kurzskala zum Corona-Verschwörungsglauben sowie zwei Einzelitems mit Bezug zu den Corona-Demonstrationen verwendet (vgl. Schließler et al. 2020; Grande et al. 2021). Der COVID-bezogene *Verschwörungsglaube*, erfasst über zwei Items, beinhaltet die Annahme, die Corona-Pandemie werde von bestimmten Gruppen genutzt, um Zwangsimpfungen durchzusetzen, sowie die Annahme, der wahre Ursprung des Virus werde „mit Absicht vor der Öffentlichkeit geheim gehalten“. Diese beiden Items werden zu einer Mittelwertskala zusammengefasst, die einen Mittelwert von 1,96 aufweist (SD = 0,98; Cronbach’s Alpha = 0,79).

Die Einstellung zu *Corona-Demonstrationen* wurde anhand des Einzelitems „Ich unterstütze die Anliegen der Corona-Demonstrationen“ erhoben. Eine diesbezügliche Handlungsbereitschaft wurde anhand des Items „Ich würde an einer Demonstration gegen die staatlichen Corona-Maßnahmen teilnehmen oder habe schon teilgenommen“ erfragt. Erneut konnte die Zustimmung bzw. Ablehnung auf einer 4‑stufigen Skala ausgedrückt werden. Die beiden Items wurden jeweils am Skalenmittelpunkt dichotomisiert und werden in den nachfolgenden Analysen als abhängige Variablen verwendet.

Als Kontrollvariablen werden Geschlecht, Alter (in 3 Kategorien) und Bildung (3-stufig) verwendet. Darüber hinaus geht die Religionszugehörigkeit (ja/nein) als Kontrollvariable in die multivariaten Analysen ein, sowie als Indikatoren für eine Ausübung religiöser Praxis zwei Variablen, die die Häufigkeit des Betens und die Häufigkeit des Besuchs eines Gotteshauses auf einer jeweils 3‑stufigen Skala (nie/gelegentlich/häufig) abbilden. Zuletzt wird auch die politische Selbsteinschätzung auf einer Skala von 1 „links“ bis 5 „rechts“ in die Analysen aufgenommen.

## Ergebnisse

### Formen der Spiritualität

Mit dem gesetzten Ziel, verschiedene Formen der Spiritualität auf den theoretisch postulierten fünf Dimensionen empirisch nachzuweisen, ist der Anspruch verbunden, dies auf möglichst sparsame Weise zu tun. Vor dem Hintergrund einer möglichen Reproduzierbarkeit und den geläufigen Platzproblemen in Erhebungsinstrumenten sollte daher zunächst die Ausgangsgröße von 42 Items reduziert werden. Alle Items zur Erfassung der Spiritualität wurden daher in mehreren explorativen Faktoranalysen auf ihre gemeinsame Passung untersucht. Nach jeder dieser Analysen wurden schrittweise diejenigen Items entfernt, die substanzielle Doppelladungen auf mehreren Faktoren aufwiesen oder für geringe Eigenwerte der Faktoren verantwortlich waren und deren Entfernung im Zusammenhang mit derartigen grenzwertigen bzw. kritischen Werten auch inhaltlich vertretbar war.

Letztlich sind insgesamt 16 Items geeignet, die Spiritualität der Befragten auf zwei Faktoren abzubilden (Tab. 1). Die abschließende Hauptkomponentenanalyse unter Einbezug dieser Items zeigt, dass alle untersuchten Items klar einem der beiden Faktoren zugeordnet werden können. Lediglich das Item „Ich bin selbst dafür verantwortlich, meinem Leben einen Sinn zu geben“ in der Dimension *Sinnempfinden* weist auf dem zweiten Faktor eine, allerdings vergleichsweise schwache, negative Nebenladung auf. Alle Faktorladungen liegen, von dieser Nebenladung abgesehen, durchgehend über 0,5. Beide Faktoren weisen zudem mit 4,44 bzw. 4,55 hohe Eigenwerte auf. Diese Faktoren zweiter Ordnung lassen sich ihrerseits wiederum in 3 inhaltliche Subdimensionen gliedern. Diese dreidimensionale Aufteilung erfolgte unter inhaltlichen Abwägungen sowie der Unterschiede in den Formulierungen bzw. Blockaufteilung der Items im Erhebungsinstrument. Die Dimensionen lauten a) *Weltbild und Ethik*, das sich auf das Zusammenleben auf der Welt und die Einbettung des Lebens in diese sowie in eine „höhere Wirklichkeit“ bezieht; b) *Wertekanon*, der die subjektive Wichtigkeit verschiedener Werte beinhaltet; und c) das *Sinnempfinden* in Bezug auf das eigene Leben.

**Table 1:** 

Item		Faktor		h^2^
		1	2	
*Weltbild und Ethik*				
Ich verstehe mein Leben als Teil der kosmischen Ordnung		–	0,574	0,455
Es gibt eine nicht erklärbare höhere Wirklichkeit		–	0,678	0,524
Es ist mir wichtig, im Einklang mit allen Lebewesen zu sein		0,635	–	0,574
Meine Seele lebt nach dem Tod weiter		–	0,740	0,564
Die Natur gibt uns vor, was richtig und falsch ist		0,535	–	0,339
Ich fühle eine tiefe Verantwortung, zu einer besseren Welt beizutragen		0,554	–	0,402
Es ist wichtig, dass sich Körper und Geist im Einklang befinden		0,648	–	0,498
*Wertekanon*				
Mitgefühl		0,719	–	0,482
Hoffnung		0,730	–	0,508
Erleuchtung		–	0,739	0,571
Harmonie		0,679	–	0,466
Verbundenheit		0,650	–	0,475
Übernatürliches		–	0,801	0,597
Erlösung		–	0,837	0,688
*Sinnempfinden*				
Ich muss mir keine Sorgen machen, weil der Sinn meines Lebens vorherbestimmt ist		–	0,622	0,377
Ich bin selbst dafür verantwortlich, meinem Leben einen Sinn zu geben		0,635	−0,417	0,400
KMO = 0,890	Eigenwert	4,441	4,550	–
N = 633	Erklärte Varianz	0,278	0,284	–

Ergebnisse der Hauptkomponentenanalyse nach Oblimin-Rotation. Faktorladungen unter 0,3 werden nicht dargestellt.

Die Items, die auf den ersten Faktor laden, zeichnen sich durch ein altruistisches Welt- und Wertebild aus, in dem Harmonie und Mitgefühl zentral sind, aber auch die Verantwortungsübernahme für eine bessere Welt eine wichtige Rolle spielt. Gleichzeitig ist eine enge Verbindung zur Natur zentral. Auch bezüglich des Sinnempfindens zeigt sich ein aktiver Charakter dieser Spiritualitätsform, die sich durch die Verantwortungsübernahme für den Sinn des eigenen Lebens auszeichnet. Insgesamt beinhaltet der Faktor vor allem solche spirituelle Komponenten, die sich auf das (Zusammen‑)Leben innerhalb der physischen Welt beziehen. Anhand dieser inhaltlichen Interpretationen wird der Faktor daher im Folgenden als *aktive Spiritualität *bezeichnet, entsprechend der Inhalte, die diese Spiritualitätsform maßgeblich strukturieren. Die zugrunde liegenden neun Items bilden einen gemeinsamen Faktor ab, der 28 % der Varianz erklärt. Mit einem Cronbach’s Alpha von 0,84 eignen sich die ausgewählten Items gut zur Bildung einer gemeinsamen Skala.

Im Gegensatz dazu beziehen sich die Items des zweiten Faktors maßgeblich auf transzendentale Inhalte wie eine höhere Wirklichkeit und die Betrachtung des eigenen Lebens als Teil der kosmischen Ordnung. Als Teil dieser kosmischen Ordnung wird auch der Sinn des eigenen Lebens als vorherbestimmt und insofern gewissermaßen unbeeinflussbar angesehen, was aber gleichzeitig die Option der Sorgenfreiheit beinhaltet. Auch der Wertekanon weist maßgeblich transzendentale Inhalte wie die Relevanz von Erleuchtung und Erlösung auf. Aufgrund dieser eher passiven (Selbst‑)Verortung der betreffenden Personen in der lebensweltlichen Realität wird dieser Faktor im Folgenden als *passive Spiritualität *bezeichnet. Wie der erste Faktor erklärt dieser etwa 28 % der Varianz. Die interne Konsistenz der darüber gebildeten Skala ist mit Cronbach’s Alpha = 0,86 als gut zu bezeichnen.

Als erstes Ergebnis kann somit zunächst festgehalten werden, dass sich die von Benesch vorgeschlagenen fünf Dimensionen der Weltanschauung empirisch im Hinblick auf lediglich drei Dimensionen nachbilden lassen. Zwar ist die erste hier bestätigte Dimension *Weltbild und Ethik *eine Kombination aus zwei der von Benesch vorgeschlagenen Dimensionen, allerdings zeigte sich bei der Übertragung in die empirische Praxis, dass Itemformulierungen kaum in der Lage sind, diese theoretisch-konzeptionelle Trennung aufrecht zu erhalten. Dies entspricht insoweit auch den weiteren eingangs diskutierten Konzeptionen und empirischen Ergebnissen, nach denen insbesondere die Sinnorientierung, das Weltbild und moralische bzw. ethische Ansichten eng miteinander zusammenhängen. Die Dimension *Menschenbild* zuletzt konnte nicht nachgebildet werden – keines der überprüften Items lud auf einem der beiden finalen Faktoren. Diese Dimension entfällt demnach bei der Konstruktion der Spiritualitätsskalen.

Insgesamt zeigt sich, dass zwei voneinander abgegrenzte Formen der Spiritualität zu identifizieren sind, die sich inhaltlich klar interpretieren lassen. Dabei werden Unterschiede zwischen diesen beiden Formen deutlich, die auf die Möglichkeit einer Differenzierung in Bezug auf die Klärung der bisherigen Ambivalenzen der Effekte von Spiritualität hindeuten. Die Skalen *aktive* und *passive Spiritualität* wurden daraus folgend als Mittelwertskalen gebildet. Zur gleichmäßigen Gewichtung der drei zugrunde liegenden Dimensionen wurden zunächst drei Subskalen gebildet, die dann erneut gemittelt wurden. Dies sorgt für einen Ausgleich der unterschiedlichen Anzahl der in die Subdimensionen und somit auch in die Gesamtskalen eingehenden Items.

**Figure 2:**
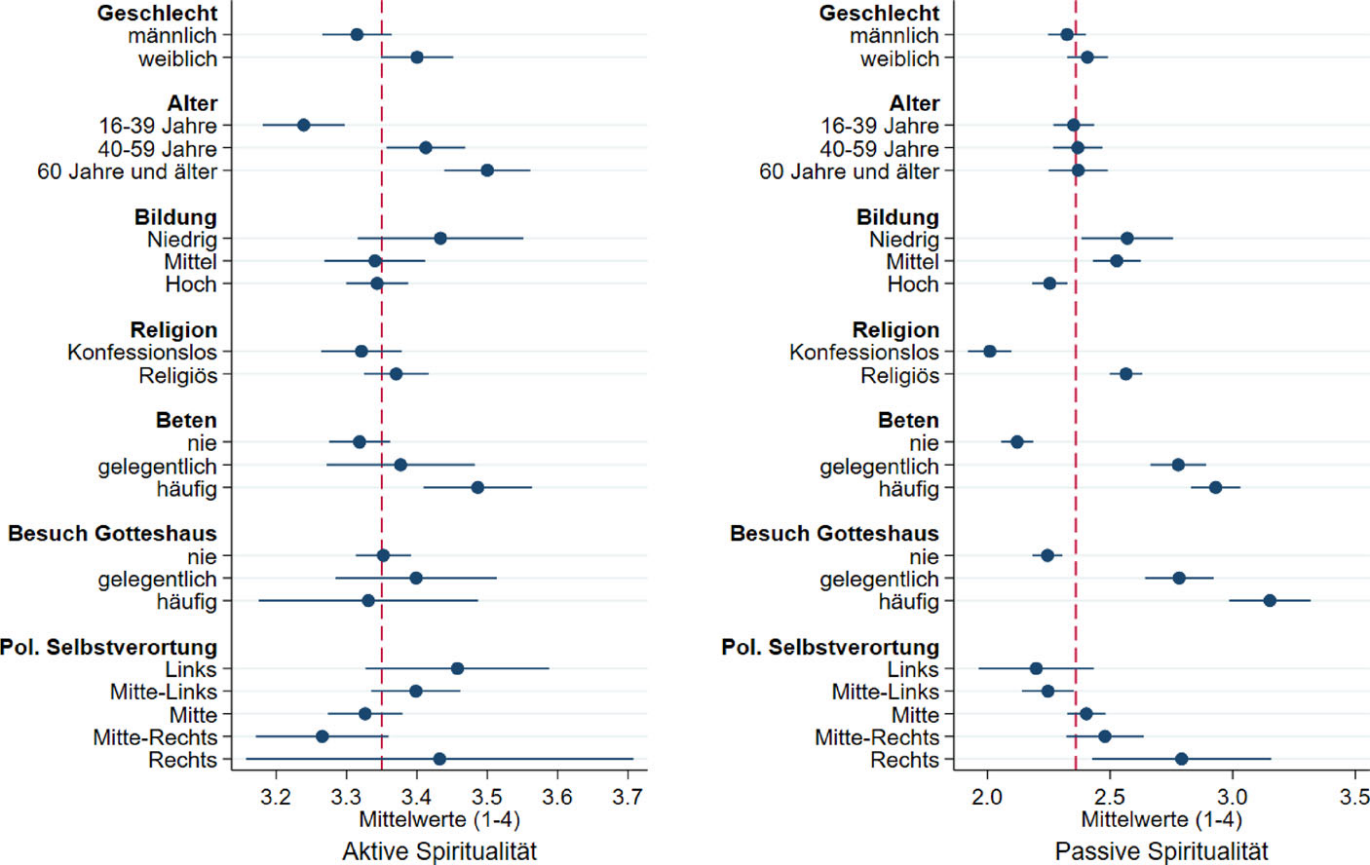

Bei einer Differenzierung der Verteilungen der aktiven und passiven Spiritualität nach gesellschaftlichen Teilgruppen (Abb. 2) zeigt sich zunächst eine generell höhere Ausprägung der aktiven (M = 3,35; SD = 0,46) als der passiven Spiritualität (M = 2,4; SD = 0,73). Bei der aktiven Spiritualität, die sich durch altruistische und harmonische Sichtweisen auszeichnet, zeigen sich leichte Geschlechterunterschiede, wobei Frauen im Durchschnitt eine höhere Ausprägung dieser Spiritualitätsform aufweisen. Im Altersvergleich ist die Ausprägung der aktiven Spiritualität umso höher, je älter die Befragten sind. Dabei liegen Personen bis 39 Jahren signifikant unter dem Gesamtdurchschnitt, Personen ab 40 Jahren hingegen darüber. In Bezug auf die Bildung der Befragten, ihre Religionszugehörigkeit und die Häufigkeit des Besuchs eines Gotteshauses lassen sich keine Unterschiede feststellen. Personen, die häufig beten, weisen hingegen eine höhere Ausprägung der aktiv-spirituellen Weltanschauung auf. Bei der politischen Selbstverortung zeigt sich tendenziell eine höhere Ausprägung der aktiven Spiritualität bei Personen, die sich links der Mitte einstufen im Vergleich zu Personen, die sich selbst in der politischen Mitte bzw. Mitte-Rechts verorten.

Ein etwas anderes Bild zeigt sich bei der Betrachtung der passiven Spiritualität. Es gibt keine Mittelwertunterschiede zwischen Männern und Frauen sowie zwischen verschiedenen Altersgruppen. Es zeigt sich ein Bildungseffekt, wobei Personen mit hoher Bildung signifikant geringere Werte aufweisen als Personen mit mittlerer oder niedriger Bildung. In Bezug auf die Religionszugehörigkeit der Befragten zeigen sich die größten Unterschiede. Personen, die keiner Religion angehören, liegen mit einem Mittelwert von 2,0 (SD = 0,69) deutlich unter dem Wert für Personen mit Religionszugehörigkeit (M = 2,6; SD = 0,67). Beide unterscheiden sich statistisch signifikant vom Gesamtmittelwert der passiven Spiritualität. Dieses Bild spiegelt sich auch in den beiden Indikatoren kirchengebundener Religiosität – Häufigkeit des Betens und des Besuchs eines Gotteshauses – wider. Je häufiger diese beiden Aktivitäten ausgeübt werden, desto höhere Mittelwerte werden auf der Skala der passiven Spiritualität erreicht. Die Ausprägung der passiven Spiritualität unterscheidet sich zudem zwischen Personen, die sich politisch eher links verorten und jenen, die sich politisch rechts verorten, wobei Personen im rechten Spektrum hier signifikant höhere Werte aufweisen. Zusammengefasst zeigt sich eine höhere Offenheit für die passive Spiritualität bei niedrig bis mittel Gebildeten, Religionszugehörigen und Personen, die ihre Religion durch Beten und Gotteshausbesuchs aktiv ausüben, sowie bei politisch eher rechts eingestellten Befragten.

Die beiden Formen der Spiritualität korrelieren nur leicht miteinander (r = 0,26; *p* < 0,001). Bei einer Dichotomisierung der beiden Skalen zeigt sich, entsprechend des zuvor festgestellten hohen Mittelwerts von 3,35, dass 85 % der Befragten einen Wert zwischen 3 und 4 auf der aktiven Spiritualität aufweisen. Nur etwa ein Viertel der Befragten (24 %) kann hingegen als in hohem Maße passiv spirituell bezeichnet werden. Von denjenigen Befragten, die hoch aktiv spirituell sind, weisen 27 % zusätzlich eine gleichermaßen hohe Ausprägung auf der passiven Spiritualität auf.

In Bezug auf den zweiten Teil der ersten Fragestellung* – Gibt es Unterschiede im Auftreten der Spiritualitätsformen zwischen Personen mit und ohne Religionszugehörigkeit? – *zeigt sich kein eindeutiges Ergebnis. Die aktive Spiritualität ist bei Personen mit und ohne Religionszugehörigkeit vergleichbar ausgeprägt. Allerdings unterscheidet sich die Ausprägung, zieht man die Häufigkeit des Betens hinzu. Deutlichere Gruppenunterschiede finden sich bei der passiven Spiritualität. Diese Form der Spiritualität, die maßgeblich transzendental bestimmt ist und Aspekte wie Erlösung, Vorherbestimmung des Lebens und den Verweis auf eine kosmische Ordnung enthält, ist bei Personen mit Religionsangehörigkeit sowie bei Personen, die häufig beten oder ein Gotteshaus besuchen, deutlich ausgeprägter. Hier lassen sich demnach die auf theoretischer Ebene genannten Inhalte von Religionen auch auf der subjektiv-spirituellen Ebene wiederfinden.

### Alternative Spiritualität: Aberglaube, Esoterik und Wissenschaftsskepsis

Auf Basis dieser Antworten auf die erste, explorative Fragestellung können die weiterführenden Forschungsfragen nun als Hypothesen dargestellt werden. Vor dem Hintergrund der erfolgten inhaltlichen Interpretation der beiden Spiritualitätsformen und den zuvor angestellten theoretischen Überlegungen zum Zusammenhang von Spiritualität als Weltanschauung und alternativen Formen der Spiritualität ist anzunehmen, dass …

- der passiv-spirituelle Glaube in einem positiven Zusammenhang mit Aberglauben, Esoterik und Wissenschaftsskepsis als Indikatoren für *alternative Spiritualität *steht (H1a).
- der aktiv-spirituelle Glaube in einem negativen Zusammenhang mit Aberglauben, Esoterik und Wissenschaftsskepsis steht (H1b).

In Bezug auf die Übertragung solcher Glaubenssätze auf gesellschaftlich und politisch relevante Haltungen und Handlungen – hier die Einstellung zu Corona-Verschwörungstheorien und zu den Corona-Demonstrationen – ist ebenfalls von gegenläufigen Effekten auszugehen. Die entsprechenden Hypothesen lauten:

- Der passiv-spirituelle Glaube erhöht den Glauben an Corona-Verschwörungstheorien und die Bereitschaft zur Teilnahme an Corona-Demonstrationen (H2a).
- Der aktiv-spirituelle Glaube verringert den Glauben an Corona-Verschwörungstheorien und die Bereitschaft zur Teilnahme an Corona-Demonstrationen (H2b).

Insofern sollten diese konträren Zusammenhänge die bisherige Ambivalenz von Spiritualität als Resilienz- vs. Risikofaktor aufschlüsseln.

Um zunächst die Zusammenhänge zwischen der aktiven und passiven Spiritualität und der alternativen Spiritualität – also Elementen von Aberglauben, Esoterik und Wissenschaftsskepsis – zu untersuchen, werden die beiden Spiritualitätsskalen in Regressionsmodellen als kontinuierliche unabhängige Variablen verwendet. Es zeigt sich (Tab. 2), dass Personen signifikant häufiger zum Aberglauben (β = 0,383; *p* < 0,001) und zum Glauben an die Wirksamkeit esoterischer Werkzeuge neigen (β = 0,525; *p* < 0,001) und zudem auch häufiger ein Misstrauen gegenüber der Wissenschaft ausdrücken (β = 0,436; *p* < 0,001), je ausgeprägter ihre passive Spiritualität ist (Modell 1). Die aktive Spiritualität hingegen (Modell 2) weist in Bezug auf die hier überprüften drei Dimensionen der alternativen Spiritualität nur einen schwachen Zusammenhang mit der Wissenschaftsskepsis auf. Dieser Zusammenhang ist knapp auf dem 5 %-Niveau signifikant. Die beiden weiteren Elemente hängen hingegen nicht mit aktiver Spiritualität zusammen.

**Table 2:** 

Modell	UV	AV1: Aberglaube	AV2: Esoterikglaube	AV3: Wissenschaftsskepsis
*M1*	Passive Spiritualität	0,383***	0,528***	0,436***
		(0,02)	(0,04)	(0,04)
*M2*	Aktive Spiritualität	−0,003	0,010	0,079*
		(0,03)	(0,07)	(0,06)

In allen Modellen wird kontrolliert für Geschlecht, Alter, Bildung, Religionszugehörigkeit und politische Selbstverortung. Es werden standardisierte beta-Koeffizienten dargestellt; Robuste Standardfehler in Klammern

*p < 0.05, **p < 0.01, ***p < 0,001

Damit lässt sich schlussfolgern, dass sich Aberglaube, Esoterik und Wissenschaftsskepsis als eigenständige Konstrukte darstellen, die mit bestimmten Facetten der Spiritualität zusammenhängen, nicht aber ein unabdingbarer Bestandteil der grundlegenden spirituellen Weltanschauung einer Person sind. Im Gegenteil zeigt sich, dass sich aktive und passive Spiritualität statistisch in Bezug auf solche Einstellungen deutlich voneinander unterscheiden. Dabei ist nur bezüglich der Wissenschaftsskepsis ein leichter Zusammenhang mit der aktiven Spiritualität festzustellen, wohingegen Personen mit einer passiv-spirituellen Weltanschauung deutlich häufiger zu allen drei Ausdrucksformen der alternativen Spiritualität neigen. Somit kann Hypothese 1a bestätigt werden, Hypothese 1b hingegen nicht, wobei zu bemerken ist, dass durch die fehlenden Effekte der aktiven Spiritualität doch eine gewisse Differenzierung zwischen den beiden Formen der Spiritualität deutlich wird. Gerade in Bezug auf die Ambivalenz der Effekte von Spiritualität lassen sich somit auch hier Hinweise auf statistisch relevante Unterschiede in Bezug auf ihre Zusammenhänge mit Formen von Aberglauben, Esoterik und Wissenschaftsskepsis erkennen.

### Der Zusammenhang mit pandemiebezogenen Haltungen

**Figure 3:**
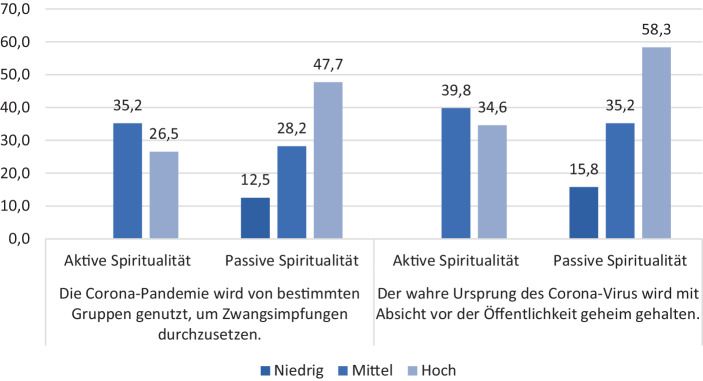

Für die Untersuchung des Zusammenhangs der spirituellen Weltanschauungen einerseits sowie Aberglaube, Esoterik und Wissenschaftsskepsis andererseits mit konkreten Inhalten im Kontext der Corona-Pandemie wird im Folgenden zunächst der Corona-bezogene Verschwörungsglaube betrachtet. Dieser wurde anhand von zwei Items erhoben, die das Virus in Verbindung mit Zwangsimpfungen und einer bewussten Geheimhaltung über seinen wahren Ursprung setzen. Im Gesamtdurchschnitt stimmen 29 % aller Befragten der Aussage zu, die Pandemie werde genutzt, um Zwangsimpfungen durchzusetzen. Mehr als ein Drittel der Befragten (35 %) vermutet eine absichtliche Geheimhaltung des Ursprungs des Virus. Es zeigt sich weiter bivariat auf deskriptiver Ebene (Abb. 3), dass die Zustimmung zur jeweiligen Verschwörungsaussage deutlich höher ausfällt, je höher die passive Spiritualität ausgeprägt ist. Die Zustimmungswerte liegen bei hoher Ausprägung dieser Spiritualitätsform bei 47,7 % bzw. 58,3 %, wohingegen bei niedriger passiver Spiritualität die Zustimmung mit 12,5 % bzw. 15,8 % substanziell geringer ausfällt. Bei der aktiven Spiritualität zeigt sich tendenziell ein umgekehrter Trend. Die Zustimmung zu den Verschwörungsaussagen liegt bei hoher aktiv-spiritueller Ausprägung bei 26,5 % bzw. 35,2 %. Die Werte sind bei mittlerer aktiver Spiritualität um 5 bis 8 Prozentpunkte höher.

Die beiden Verschwörungsitems korrelieren mit r = 0,66 miteinander. Sie werden in einer Kurzskala „Verschwörungsglaube“ zusammengefasst und gehen in den weiteren Analysen als kontinuierliche abhängige Variable in hierarchische lineare Regressionen ein (Tab. 3). Die Modelle 1 bis 3 stellen die Einflüsse der verwendeten Kontrollvariablen dar. In Modell 1 zeigt sich nur ein Bildungseffekt, wobei Personen mit Abitur signifikant seltener an Verschwörungserzählungen glauben als Personen mit einer niedrigeren Bildung. Die Religionszugehörigkeit (Modell 2) hat alleinstehend keinen Einfluss auf den Verschwörungsglauben, zieht man jedoch Indikatoren kirchengebundener Religiosität heran, zeigen sich leicht verstärkende Effekte der Häufigkeiten des Betens und des Besuchs eines Gotteshauses auf das Ausmaß des Verschwörungsglaubens. Die politische Selbstverortung der Befragten (Modell 3) weist ebenfalls einen Einfluss auf – je weiter sich eine Person im rechten Spektrum verortet, desto eher tendiert sie auch zu einem Verschwörungsglauben.

**Table 3:** 

	M1	M2	M3	M4	M5
*Geschlecht* *(1 = weiblich)*	−0,022	−0,010	0,004	−0,000	−0,042
	(0,08)	(0,08)	(0,08)	(0,07)	(0,07)
*Alter (Ref.* *=* *16–39 Jahre)*					
40–59 Jahre	−0,048	−0,037	−0,033	−0,013	−0,064
	(0,09)	(0,09)	(0,08)	(0,08)	(0,07)
60 Jahre und älter	−0,059	−0,038	−0,015	0,012	−0,023
	(0,11)	(0,12)	(0,11)	(0,11)	(0,10)
*Bildung* *(1 = Abitur)*	−0,220***	−0,212***	−0,192***	−0,133**	−0,071
	(0,09)	(0,09)	(0,08)	(0,08)	(0,08)
*Religionszugehörigkeit*	–	0,016	−0,009	−0,054	0,001
*(1 = ja)*	–	(0,09)	(0,09)	(0,08)	(0,08)
*Häufigkeit Beten (Ref. = nie)*					
Gelegentlich	–	0,127*	0,120*	0,051	0,034
	–	(0,14)	(0,14)	(0,14)	(0,12)
Häufig	–	0,047	0,035	−0,036	−0,010
	–	(0,15)	(0,14)	(0,14)	(0,13)
*Gotteshausbesuch (Ref. = nie)*					
Gelegentlich	–	0,032	0,055	0,040	0,017
	–	(0,16)	(0,15)	(0,15)	(0,13)
Häufig	–	0,114*	0,111*	0,066	0,036
	–	(0,19)	(0,18)	(0,18)	(0,16)
*Politische Selbstverortung*	–	–	0,247***	0,207***	0,167***
	–	–	(0,04)	(0,04)	(0,04)
*Aktive Spiritualität*	–	–	–	−0,105**	−0,082*
	–	–	–	(0,08)	(0,08)
*Passive Spiritualität*	–	–	–	0,328***	0,090
	–	–	–	(0,06)	(0,07)
*Aberglaube*	–	–	–	–	0,034
	–	–	–	–	(0,02)
*Esoterikglaube*	–	–	–	–	0,082
	–	–	–	–	(0,06)
*Wissenschaftsskepsis*	–	–	–	–	0,414***
	–	–	–	–	(0,06)
*N*	607	607	607	607	607
Adj. *R*^2^	0,04	0,07	0,13	0,20	0,35

Es werden standardisierte beta-Koeffizienten dargestellt; robuste Standardfehler in Klammern

*p < 0.05, **p < 0.01, ***p < 0,001

Modell 4 zeigt sodann, dass die aktive und passive Spiritualität gegenteilige Effekte auf den Glauben an Verschwörungserzählungen haben. Während der negative Koeffizient der aktiven Spiritualität auf einen Schutzfaktor hinweist (β = −0,105; *p* < 0,01), hängt ein passives spirituelles Weltbild positiv mit dem Glauben an die beiden Verschwörungserzählungen zusammen (β = 0,328; *p* < 0,001). Der Einfluss der Kontrollvariablen besteht nun nur noch bezüglich der Bildung sowie der politischen Selbstverortung der Befragten. Unter Hinzunahme der alternativen Spiritualität im Sinne von Aberglauben, Esoterikglauben und Wissenschaftsskepsis in Modell 5 werden die Effekte der aktiven Spiritualität schwächer, weisen jedoch immer noch in Richtung eines Schutzeffektes vor dem Glauben an Verschwörungsinhalte. Die passive Spiritualität weist nun keinen Einfluss mehr auf. Während von den alternativ-spirituellen Elementen der Aberglaube und der Glaube an die Wirksamkeit esoterischer Werkzeuge keinen Einfluss auf die Zustimmung zu den Verschwörungserzählungen hat, weist die Wissenschaftsskepsis hier die stärksten Zusammenhänge auf (β = 0,414; *p* < 0,001). Mit 35 % Varianzaufklärung kann das Gesamtmodell substanzielle Anteile zur Erklärung eines COVID-Verschwörungsglaubens beitragen.

Um diese Zusammenhänge zwischen spirituellen und verschwörungstheoretischen Einstellungen auf die Ebene der Handlungsbereitschaft zu übertragen, werden im Folgenden die Wirkungen der bisherigen Konstrukte auf die Unterstützung der Corona-Demonstrationen einerseits sowie die Bereitschaft zur Teilnahme an solchen Demonstrationen andererseits untersucht. Die beiden abhängigen Variablen gehen in dichotomisierter Form in logistische Regressionsmodelle ein (Tab. 4).

**Table 4:** 

	Unterstützung der Corona-Demonstrationen			Bereitschaft zur Teilnahme an Corona-Demonstrationen		
	M1a	M2a	M3a	M1b	M2b	M3b
Aktive Spiritualität	0,519**	0,530*	0,632	0,307***	0,307***	0,333**
	(0,13)	(0,14)	(0,19)	(0,08)	(0,09)	(0,11)
Passive Spiritualität	3,351***	1,645*	1,442	4,319***	1,925*	1,671
	(0,63)	(0,35)	(0,38)	(0,92)	(0,50)	(0,48)
Aberglaube	–	1,077	1,059	–	1,059	1,027
	–	(0,08)	(0,09)	–	(0,08)	(0,09)
Esoterikglaube	–	1,372	1,359	–	1,575*	1,613*
	–	(0,25)	(0,28)	–	(0,30)	(0,35)
Wissenschaftsskepsis	–	3,586***	1,753*	–	3,434***	1,680*
	–	(0,73)	(0,41)	–	(0,76)	(0,44)
COVID-Verschwörungsglaube	–	–	3,813***	–	–	3,816***
	–	–	(0,61)	–	–	(0,67)
*N*	600	600	600	605	605	605
Pseudo *R*^2^	0,17	0,26	0,40	0,17	0,26	0,39

In allen Modellen wird kontrolliert für Geschlecht, Alter, Bildung, Religionszugehörigkeit, Häufigkeit des Betens, Häufigkeit des Gotteshausbesuchs und politische Selbstverortung. In den Gesamtmodellen kann nur für die politische Selbstverortung sowie ein häufiger Besuch eines Gotteshauses ein signifikanter Effekt nachgewiesen werden (vgl. Tab. 3). Aus Gründen der Übersichtlichkeit wird daher auf die Darstellung dieser Variablen verzichtet. Es werden Odds Ratios dargestellt; Standardfehler in Klammern

*p < 0.05, **p < 0.01, ***p < 0,001

In beiden Modellblöcken zeigen sich ähnliche Effekte, wie sie auch schon in Bezug auf den Verschwörungsglauben festgestellt werden konnten. Die Modelle 1a und 1b zeigen erneut die gegenteiligen Effekte der aktiven und der passiven Spiritualität auf, wobei aktiv Spirituelle die Corona-Demonstrationen seltener unterstützen (OR = 0,519; *p* < 0,01) und auch deutlich seltener eine Teilnahmebereitschaft signalisieren (OR = 0,307; *p* < 0,001). Passiv Spirituelle hingegen weisen eine um jeweils den Faktor 3,4 bis 4,3 erhöhte relative Chance auf, die Demonstrationen zu unterstützen (OR = 3,351; *p* < 0,001) bzw. selbst an ihnen teilzunehmen (OR = 4,319; *p* < 0,001).

Die Zusammenhänge mit der Demonstrationsunterstützung werden in Bezug auf die beiden Spiritualitätsformen geringer, wenn Aberglaube, Esoterik und Wissenschaftsskepsis in die Modelle aufgenommen werden (Modell 2a). Die Wissenschaftsskepsis weist hier nun – wie schon in der vorherigen linearen Analyse – einen stark positiven Zusammenhang mit der abhängigen Variablen auf (OR = 3,586; *p* < 0,001), während sich der Effekt der passiven Spiritualität gleichzeitig halbiert und nur noch auf dem 5 %-Niveau signifikant ist (OR = 1,645; *p* < 0,05).^2^ Unter Hinzunahme des Verschwörungsglaubens (Modell 3a) verschwinden die Effekt der aktiven und passiven Spiritualität vollständig. Der Einfluss der beiden Spiritualitätsformen kann augenscheinlich komplett über den Einfluss des Verschwörungsglaubens erklärt werden, wie es schon die vorherigen linearen Modelle nahelegten.

In Bezug auf die Teilnahmebereitschaft sind gleichartige Zusammenhänge festzustellen. Auch hier wird der ursprüngliche Risikoeffekt der passiven Spiritualität unter Hinzunahme der drei Komponenten der alternativen Spiritualität mehr als halbiert (Modell 2b). Die Wissenschaftsskepsis ist in einer ähnlichen Höhe wie zuvor der stärkste Prädiktor für die Demonstrationsbereitschaft (OR = 3,434; *p* < 0,001). In Modell 3b verschwindet der Einfluss der passiven Spiritualität wiederum unter Hinzunahme des COVID-Verschwörungsglaubens komplett. Anders als in der Frage nach der Unterstützung von Corona-Demonstrationen besteht zudem auch im Gesamtmodell ein leicht signifikanter Effekt des Esoterikglaubens. Der negative, insofern schützende, Einfluss der aktiven Spiritualität ist in allen drei Modellen gleichermaßen stark ausgeprägt und hochsignifikant.

Bezüglich der dritten Fragestellung dieses Beitrags zeigt sich somit zusammenfassend, dass die aktive Spiritualität übergreifend als Schutzfaktor fungiert und damit konträr zur passiven Spiritualität verläuft, die sowohl den Verschwörungsglauben als auch die Unterstützung von und Teilnahmebereitschaft an Corona-Demonstrationen erhöht. Die in diesem Kontext formulierten Hypothesen 2a und 2b können insofern zunächst bestätigt werden. Es wird aber auch sichtbar, dass die Annahmen dieser Hypothesen in weiterführenden Analysen nicht durchgehend weiter aufrecht erhalten werden können, da insbesondere die passive Spiritualität unter Einbezug der esoterischen und wissenschaftsskeptischen Einstellungen an Einfluss verliert. Der Aberglaube hingegen stellt in keinem der überprüften Modelle einen Einflussfaktor dar. Vielmehr zeigt sich ein starker Einfluss von wissenschaftsskeptischen Elementen und dem Glauben an Corona-bezogene Verschwörungsnarrative.

## Diskussion und Zusammenfassung

In diesem Beitrag wurden empirische Fragen aufgeworfen, die sich mit der Rolle spiritueller Überzeugungen von Personen und deren Einfluss auf den Glauben an Verschwörungserzählungen und die Protestbereitschaft im Kontext der Corona-Pandemie befassen. Das Zusammenspiel mit Einstellungen aus dem esoterischen Bereich, die im Rahmen solcher Demonstrationen vermehrt zum Ausdruck kommen, sollte auf diese Weise aufgeschlüsselt werden. Hierbei wurden spirituelle Weltanschauungen in einer Erweiterung bisheriger Untersuchungen unabhängig von religiösen Ansichten und Zugehörigkeiten erfasst, was den Einbezug von Menschen ohne religiöse Zugehörigkeit oder institutionelle Bezüge erlaubte.

Fragestellung 1 befasste sich mit der Frage der Möglichkeit einer differenzierten und zugleich möglichst effizienten Messung von Spiritualität unter Aufgreifen der diesbezüglich theoretisch für relevant erachteten Dimensionen. Es ließen sich faktoranalytisch zwei Formen der Spiritualität identifizieren, die jeweils Aspekte von drei theoretisch relevanten Subdimensionen der Spiritualität in unterschiedlichen Konfigurationen enthielten. Diese beiden Formen, für welche die Bezeichnung als *aktive* und *passive Spiritualität* vorgeschlagen wird, weisen unterschiedliche Bezüge auf. Die aktive Spiritualität enthält maßgeblich altruistische und moralisch-ethische Elemente, die eine Verantwortung für andere Menschen und eine Verbindung mit der Welt und Natur ausdrücken. Die passive Spiritualität hingegen hat einen stärker individualbezogenen, egozentrischen Bezug, innerhalb dessen sich die Personen als Teil eines vorherbestimmten Universums verstehen, auf (persönliche) Erleuchtung und Erlösung abzielen und transzendentale Inhalte in den Mittelpunkt ihrer Weltanschauung rücken.

Die Verteilungen dieser beiden Formen der Spiritualität zeigen, dass aktiv-spirituelle Haltungen in der Bevölkerung sehr weit verbreitet sind. Bezüglich der passiven Spiritualität, die seltener auftritt, findet sich zudem ein großer Unterschied zwischen Personen mit und ohne Religionszugehörigkeit, was so für die aktive Spiritualität nicht gilt. Hier scheint eine enge Verbindung zwischen religiösen und den hier gemessenen spirituellen Elementen vorzuliegen, die letztlich auch einen Hinweis auf die Art der Kontingenzbewältigung geben können: „Durch das Bestehen auf eine determinierende Ordnung verlieren moralische Fragen an Ambiguität und jede persönliche oder soziale Erfahrung wird als „sinnvoll“ gedeutet“ (Steppacher 2021, S. 92). Eine solche determinierende Ordnung wird in den Inhalten der passiven Spiritualität auf allen Subdimensionen besonders deutlich.

Im Rahmen der Fragestellungen 2 und 3 konnte sodann aufgezeigt werden, dass diese beiden Spiritualitätsformen unterschiedliche Zusammenhänge mit alternativ-spirituellen Elementen (Aberglauben, Esoterik und Wissenschaftsskepsis) aufweisen und darüber hinaus gegenteilige Effekte auf den Glauben an Verschwörungserzählungen zeigen. Dies lässt sich in Einklang bringen mit den eingangs dargestellten Verknüpfungen von Elementen der Sinnsuche, esoterischen Ausdrucksformen und Verschwörungserzählungen: „[…] Daher ist wichtiger als die Frage, *wer* eigentlich dahintersteckt, die Behauptung, *dass* jemand dahintersteckt. Wichtig ist, dass nichts ohne Grund passiert, sekundär ist hingegen, was genau dieser Grund ist“ (Schäfer und Frei 2021, S. 400, Hervorhebung im Original). Hier wird die Verbindung zwischen Kontingenzbewusstsein und spirituellem Sinnempfinden sichtbar.

Inwiefern bestimmte Inhalte der Spiritualität zu verschiedenen Ausprägungen des Verschwörungsglaubens führen, z. B. in Abhängigkeit der innerhalb der Erzählung verantwortlich gemachten Gruppe, konnte in den vorliegenden Analysen nicht weiter aufgegriffen werden. Dies wäre indessen ein fruchtbares Thema für die weitere Forschung. Die Analyse dieser Frage könnte dazu beitragen, unterschiedliche Formen der Ablehnung bestimmter gesellschaftlicher Akteure (im Sinne einer gruppenbezogenen Intoleranz), sowie die Ablehnung politischer Akteure und Einrichtungen näher zu beleuchten.

Die Ergebnisse zeigen zunächst auf methodischer Ebene, dass eine Erfassung unterschiedlicher Spiritualitätsformen religionsunabhängig möglich ist und diese eindeutig interpretierbar sind. Die Messung der Spiritualität, wie sie hier vorgenommen wurde, bietet dabei Vorteile gegenüber der bisherigen Messungen anhand der spirituellen und religiösen Selbsteinschätzung von Personen, da sie subjektive Einstellungen und Weltinterpretationen klarer einbezieht. Dies ermöglicht gleichzeitig den Rückbezug auf theoretische Modelle, die neben ‚objektiverten‘ Elementen auch Aspekte von u. a. Sinnorientierung, Werten und ethischen Belangen beinhalten. Auf diese Weise können zudem auch Personen ohne dezidierte Konfessionszugehörigkeit in Untersuchungen einbezogen werden. Gerade aufgrund der steigenden Anzahl konfessionsloser Menschen in Deutschland bei gleichzeitig größer werdenden Sichtbarkeit spiritueller Haltungen und Ausdrucksformen erscheint dies sehr relevant.

In Bezug auf diese Spiritualitätsformen erscheint es sinnvoll, in zukünftigen Untersuchungen eine Verortung von Personen im Rahmen clusteranalytischer Verfahren durchzuführen, um die hier gezeigten Überschneidungen von aktiver und passiver Spiritualität zu vermeiden und klare Bezüge von spirituellen Gruppierungen weiter zu untersuchen. Auch wäre es auf diese Weise besser möglich, subjektive Unterschiede in der Relevanz einzelner Dimensionen bzw. Inhalte der Spiritualität herauszustellen. Eine solche Gewichtung wurde hier nicht vorgenommen. Vielmehr wurde hier auf explorativer Basis ein Messinstrument vorgestellt, auf dessen Grundlage derartige Untersuchungen möglich wären. Vor diesem Hintergrund muss auch auf die Einschränkung der hier verwendeten Stichprobe verwiesen werden, anhand derer die Ergebnisse mit Blick auf die Verbreitung der entsprechenden spirituellen Haltungen nicht verallgemeinerungsfähig auf die Allgemeinbevölkerung sind. Insoweit wäre es wünschenswert, wenn das hier entwickelte Messinstrument auch in künftigen bevölkerungsrepräsentativen Studien zum Einsatz kommen könnte.

Auf inhaltlicher Ebene wird anhand dieser Untersuchung deutlich, dass den ambivalenten Einflüssen von Spiritualität, wie sie eingangs diskutiert wurden, verschiedene Konfigurationen spiritueller Haltungen und damit verschiedene Weltanschauungen zugrunde liegen, die bisher nicht immer eindeutig verortet werden konnten. Hierbei hängt die passive Spiritualität eng mit parapsychologischen Ausdrucksformen im Sinne von Aberglauben und Esoterik zusammen. Auch die Wissenschaftsskepsis, u. a. durch die Ablehnung von ‚Schulmedizin‘ ausgedrückt, spielt hier eine zentrale Rolle. Das Zusammenspiel dieser Elemente, wie sie im Rahmen der „contaminated mindware“ (Rizaq et al. 2021) angenommen wird, kann insofern hier ebenfalls aufgezeigt werden. Dabei ist allerdings auch festzustellen, dass abergläubische Inhalte im Vergleich zu den anderen beiden Elementen der alternativen Spiritualität keinen Einfluss auf verschwörungstheoretische und pandemiebezogene Einstellungen haben. Während einige Untersuchungen hier zu anderen Schlüssen kommen, zeigte sich ein ähnliches Bild bereits in der Leipziger Autoritarismus Studie (Schließler et al. 2020), in deren Untersuchung der Aberglaube ebenfalls den schwächsten Einfluss in entsprechenden Modellen aufweist. Der Verschwörungsglaube scheint sich demnach eher aus esoterischen und wissenschaftsskeptischen Einstellungen von Personen zu speisen. Vor diesem Hintergrund sollte überprüft werden, ob andere Messungen von Aberglauben – dessen Abgrenzung zu Esoterikglauben schon auf definitorischer Basis nicht unproblematisch ist – besser geeignet wäre, die tatsächlichen Inhalte empirisch abzubilden. Auch die Skala zur Erfassung der Wissenschaftsskepsis könnte weiter ausgebaut werden, wobei auch hier darauf geachtet werden sollte, zu große Überschneidungen mit esoterischen Elementen zu vermeiden, um eine trennscharfe Erfassung zu gewährleisten. Nichtsdestotrotz kann anhand dieser Ergebnisse ein empirischer Hinweis auf die Gültigkeit des Conspirituality-Begriffs, der esoterische und verschwörungstheoretische Komponenten in sich vereint, gegeben werden.

Die Relevanz dieses Forschungsbereichs ergibt sich im Kontext der Corona-Pandemie nicht zuletzt daraus, dass anhand diverser aktueller Studien zu diesem Thema berichtet wird, dass bei Personen mit bestimmten esoterischen und verschwörungstheoretischen Haltungen ein Hang zu politisch rechten Einstellungen besteht. Dieser scheint auch innerhalb der Demonstrationen gegen die Corona-Maßnahmen einen immer größeren Platz einzunehmen (vgl. Grande et al. 2021) und kann auf diese Weise auch das gesellschaftliche und politische System vor weitere Herausforderungen stellen. Schon in Bezug auf die passive Spiritualität zeigte sich eine höhere Verbreitung unter Personen, die sich selbst im eher rechten Spektrum verorten und in den darauf aufbauenden Analysen konnten Einflüsse der politischen Selbstverortung in Bezug auf den Verschwörungsglauben festgestellt werden, die unter Einbezug spiritueller Elemente zwar geringer wurden, letztlich aber weiterhin einen eigenständigen Effekt hatten.

Derartige Überlappungen der Weltanschauungen sowie der alternativen Spiritualität mit Verschwörungserzählungen in Bezug auf deren Inhalte und anhand dessen vorgenommener (Selbst‑)Rechtfertigungen lassen darauf schließen, dass auch im Bereich der Extremismusforschung ein Blick auf die Gruppe nicht-religiöser Spiritueller geworfen werden sollte, wie weiter oben schon angedeutet wurde. So sind Verschwörungsmythen häufig inhärenter Bestandteil eines Radikalisierungsprozesses, wenn sie nicht selbst bereits extremistische Positionen beinhalten: „Extremistische Einstellung und Verschwörungstheorien sind zwei unterschiedliche Bausteine einer destruktiven Entwicklung“ (Endrass et al. 2021, S. 116). Somit ist eine empirische Verbindung dieser Phänomene wünschenswert, die bisher beispielsweise schon im Rahmen von Untersuchungen zu Religionszugehörigkeit und AfD-Präferenz bzw. Rechtsextremismus (Siegers und Jedinger 2020; Schneider et al. 2021) durchgeführt wurden. Diese könnten anhand der hier aufgezeigten Möglichkeiten der religionsneutralen Messung spiritueller Ansichten weiter ausdifferenziert werden. In einem damit zusammenhängenden Schritt wäre es aber im Gegenteil auch möglich, derartige spirituelle Ansichten explizit in Verbindung mit der Religionszugehörigkeit zu untersuchen. Da eine höhere Ausprägung der passiven Spiritualität unter Personen mit Religionszugehörigkeit sowie Personen mit einer ausgeprägten religiösen Praxis festgestellt werden konnte, könnten entsprechende Forschungsansätze eine Verbindung von spirituellem, religiösen und Verschwörungsglauben aufzeigen, aus dem möglicherweise Präventionsansätze zu Verschwörungsglauben und seinen Folgen, fokussiert auf relevante (religiöse) Subgruppen, resultieren könnten (Arzheimer und Carter 2009).

Auch religiöser Extremismus könnte auf diese Weise klarer umfasst werden, innerhalb dessen ein Fokus auf verschiedene Dimensionen der (religiösen) Weltanschauung zentral zur Erklärung seiner Entstehung und Ausprägung ist. Es ist davon auszugehen, dass religiöser Extremismus nicht allein auf Faktoren wie der Zugehörigkeit zu einer Religion oder der Häufigkeit des Betens beruht, sondern dass dieser ebenfalls ein mehrdimensionales Phänomen ist, in dem einzelne religiöse Werte und Überzeugungen ausschlaggebend für die Ausbildung von fundamentalistischen bis hin zu extremistischen Haltungen sind (vgl. z. B. Brettfeld und Wetzels 2007).

Insgesamt können solche thematischen Bezüge dazu beitragen, Elemente des gesellschaftlichen Zusammenhalts im Kontext einer sozialen Krisensituation in Bezug zu bestimmten Weltanschauungen zu setzen. Auf dieser Basis erscheint es möglich und erstrebenswert, Unterschiede und Gemeinsamkeiten entsprechender Gruppen, deren Argumentationsmuster und nicht zuletzt deren (politische) Ziele näher zu untersuchen, aufzuschlüsseln und relevante Erklärungsmechanismen aufzuzeigen.

## Footnotes

1 Alle nachfolgenden Analysen wurden zusätzlich auch unter Ausschluss dieser Kategorie durchgeführt. Es konnten keine maßgeblichen Unterschiede in den Effekten der Skala festgestellt werden.

2 Es wurden zusätzlich Interaktionsterme der beiden Formen der Spiritualität mit jeweils allen Komponenten der alternativen Spiritualität in die Analysemodelle eingefügt. Es konnten keine Interaktionseffekte festgestellt werden. Gleiches gilt für die Bereitschaft zur Teilnahme an Corona-Demonstrationen.
